# Assessment of right ventricular endocardial fibroelastosis in fetuses with critical pulmonary stenosis and pulmonary atresia with intact ventricular septum

**DOI:** 10.3389/fped.2024.1518898

**Published:** 2025-01-10

**Authors:** Yue Wang, Gang Luo, Yi Sun, Taotao Chen, Silin Pan

**Affiliations:** ^1^ Heart Center, Women and Children’s Hospital, Qingdao University, Qingdao, China; ^2^ Department of Obstetric Ultrasound, Women and Children’s Hospital, Qingdao University, Qingdao, China

**Keywords:** right ventricle, endocardial fibroelastosis, critical pulmonary stenosis, pulmonary atresia with intact ventricular septum, fetal echocardiogram

## Abstract

**Background:**

This study aimed to assess right ventricular (RV) endocardial fibroelastosis (EFE) in fetuses with critical pulmonary stenosis (CPS) and pulmonary atresia with intact ventricular septum (PA-IVS) and to investigate the implications of RV EFE for circulatory outcomes.

**Methods:**

Fetal echocardiographic data from July 2018 to January 2021 were collected. Three reviewers independently graded EFE based on the presence and extent of endocardial echogenicity. Since this is a novel study on grading RV EFE, intra- and interobserver comparisons were performed. The associations among EFE severity, anatomic variables, and late-gestational circulatory outcomes were analysed.

**Results:**

Eighty-one patients with RV EFE were identified. By consensus, EFE severity was assessed as Grade 1 (*n* = 66, 81.5%) or Grade 2 (*n* = 15, 18.5%). At the first consultation, RV sphericity values were greater in Grade 2 EFE fetuses than in Grade 1 EFE fetuses, implying more severe noncompliance and worse diastolic function. From the first consultation to late gestation, significant differences were observed in the changes in the tricuspid/mitral valve (TV/MV) annulus diameter (*P* = 0.042) and TV *z*-score (*P* = 0.001) between the Grade 1 and Grade 2 RV EFE groups. Among the ten patients who underwent fetal cardiac intervention (FCI), the restoration of the TV *z*-score was more significant in Grade 2 RV EFE fetuses than in Grade 1 EFE fetuses. Among Grade 2 EFE cases, fetuses who underwent FCI exhibited greater changes in the right/left ventricular (RV/LV) long-axis dimension, TV/MV, and RV sphericity compared to non-FCI fetuses, indicating that FCI benefited Grade 2 EFE fetuses by restoring the development of ventricular structure.

**Conclusions:**

This study graded RV EFE in fetuses with CPS/PA-IVS, shedding light on its implications for circulatory outcomes. FCI offered benefits in Grade 2 RV EFE patients, suggesting its potential to preserve cardiac development in affected fetuses with CPS/PA-IVS.

## 1 Introduction

Pulmonary atresia with intact ventricular septum (PA-IVS) is a rare form of complex congenital heart disease. Critical pulmonary stenosis (CPS) resembles the favourable extreme circumstance of PA-IVS. Both CPS and PA-IVS are severe right ventricular outflow tract (RVOT) obstructions that have analogous hemodynamic alterations (1). Associated neonates require repeated postnatal catheter intervention and surgery and struggle to achieve biventricular circulation (2), resulting in an extremely poor prognosis.

To optimize the conditions for right ventricle (RV) and valve growth *in utero*, RVOT obstruction needs to be eliminated. Successful fetal cardiac intervention (FCI) can relieve RVOT obstruction and promote continued growth of the RV during the prenatal period, increasing the probability of a biventricular outcome. Patients with CPS/PA-IVS have been reported to have improved postnatal prognoses following FCI (3–7). However, the indications for FCI in patients with CPS/PA-IVS remain debatable. Due to the limited population, restricted examination technology, ethical considerations, and other factors, pathophysiologic variables are rarely assessed before FCI.

Endocardial fibroelastosis (EFE) has been proven to hinder pre- and postnatal heart development. As the ventricle becomes more fibrotic and hypertrophic, compliance deteriorates, leading to worsened ventricular diastolic dysfunction (8–10). However, due to the notable heterogeneity and small population, there are no published data available regarding the reliability of systematically grading RV EFE in fetuses with CPS/PA-IVS.

It was assumed that severe RV EFE might substantially influence the circulatory outcomes of CPS/PA-IVS. The current study aimed to grade RV EFE severity in the CPS/PA-IVS and investigate its relationship with morphological parameters, thus providing a more comprehensive assessment for selecting FCI candidates.

## 2 Methods

### 2.1 Study cohort

This retrospective single-center cohort study included fetuses with CPS/PA-IVS from July 2018 to January 2021. A total of 178 consecutive patients were included. CPS was defined as pulmonary valve dysplasia (valve hyperechogenicity, thickening or hypomobility) combined with systolic flow acceleration (peak velocity >1.4 m/s) with reversed flow in the ductus arteriosus (11, 12). PA was defined as the absence of flow across the pulmonary valve (11, 12). After excluding patients who ceased gestation (*n* = 78, 43.8%) or lacked follow-up records (*n* = 12, 6.7%), 88 echocardiographic recordings were reviewed.

Each participant's legal guardian provided written informed consent prior to enrolling in the study. All studies were conducted in accordance with both the Declaration of Helsinki and the Declaration of Istanbul.

### 2.2 Analysis of echocardiographic images

The International Society of Ultrasound in Obstetrics and Gynecology guidelines were followed for all fetal echocardiography (13). Two fetal echocardiographers and an experienced cardiologist who were unaware of the outcome or whether the fetuses had undergone FCI reviewed the echocardiographic images.

The ventricular and valvular dimensions, including the RV long-axis dimension, RV short-axis dimension, left ventricular (LV) long-axis dimension, pulmonary valve (PV) annulus diameter, aortic valve (AV) annulus diameter, tricuspid valve (TV) annulus diameter, and mitral valve (MV) annulus diameter, were measured. The TV *z*-score was calculated according to reports by Krishnan et al. (14). Right ventricular sphericity was estimated as the ratio of short-axis to long-axis dimensions.

### 2.3 RV EFE severity assessment

It was assumed that RV EFE could substantially impact circulatory outcomes. Due to the absence of an established grading standard for RVOT obstruction diseases, we strived to systematically assess RV EFE. EFE was repeatedly assessed mainly on the basis of echocardiographic findings of the presence, extent, and range of endocardial brightness (e.g., echogenicity) in the RV.

**Figure 1:**
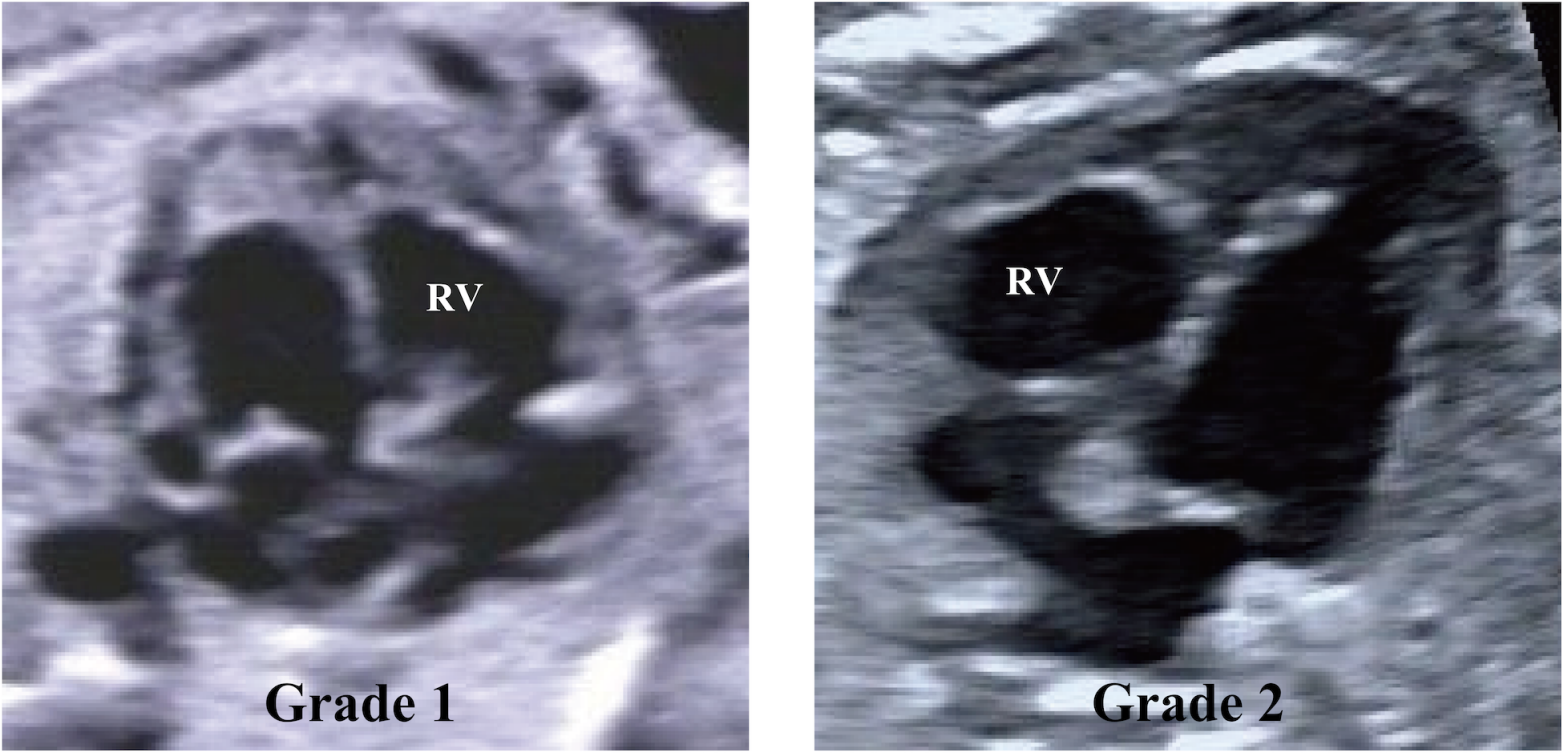
Echocardiographic images demonstrating examples of right ventricle (RV) endocardial fibroelastosis grades. Grade 1, scattered echogenic spots within the RV. Grade 2, echogenic patches throughout the RV.

In the current study, the grading of RV EFE primarily relies on the expertise of echocardiography reviewers and the assessment criteria used for LV EFE in hypoplastic left heart syndrome (HLHS) (15). RV EFE severity in CPS/PA-IVS patients was graded as follows: (1) scattered echogenic spots within the RV; (2) echogenic patches throughout the RV. Figure 1 and Supplementary Figure 1 show examples of each RV EFE grade.

In detail, Grade 1 was defined as scattered, punctate echogenic regions characterized by discrete, well-demarcated foci of increased echogenicity. These regions were small, non-confluent, and exhibited a rounded or slightly irregular shape, without forming continuous segments. Grade 2 was defined as larger, confluent echogenic regions with a patchy or band-like appearance. These areas were characterized by continuous or coalescent patterns of echogenicity, involving broader portions of the RV endocardium. Grade 2 appearance was elongated or composed of contiguous clusters, indicative of more extensive fibrosis or endocardial involvement.

### 2.4 Intra- and interobserver variability

The intra- and interobserver reproducibility of RV EFE gradation, septal EFE severity, and relative RV length were assessed by weighted kappa and Fleiss kappa analysis (Table 1). To assess intraobserver variability, the measurements made by each reviewer were compared with each other. To assess interobserver variability, the two sets of assessments made by each reviewer were compared.

**Table 1:** Intra- and interobserver variability in right ventricular (RV) endocardial fibroelastosis (EFE) assessment.

Variable	Kappa or weighted kappa	Standard error
Intraobserver variability		
RV EFE severity grade (0–2)		
Reviewer 1	0.736	0.064
Reviewer 2	0.761	0.060
Reviewer 3	0.639	0.079
Septal EFE severity (0–2)		
Reviewer 1	0.928	0.928
Reviewer 2	0.914	0.038
Reviewer 3	0.923	0.038
Septal EFE more severe than elsewhere (yes or no)		
Reviewer 1	0.801	0.077
Reviewer 2	0.537	0.108
Reviewer 3	0.810	0.082
Interobserver variability^a^		
RV EFE severity grade (0–2)	0.648	0.044
Septal EFE severity (0–2)	0.693	0.051
Septal EFE more severe than elsewhere (yes or no)	0.650	0.064
Interobserver variability^b^		
RV EFE severity grade (0–2)	0.567	0.044
Septal EFE severity (0–2)	0.632	0.050
Septal EFE more severe than elsewhere (yes or no)	0.592	0.064

^a^ The first set of assessments by the three reviewers was compared.

^b^ The second set of assessments by the three reviewers was compared.

### 2.5 FCI procedure

In CPS/PA-IVS, the FCI method is fetal pulmonary valvuloplasty under ultrasound guidance. The procedures were previously described in detail (16, 17). In our hospital, FCI was considered in the following situations: membranous PA, an intact ventricular septum, and hypoplastic valves with a visible but relatively small RV. The exclusion criteria included muscular atresia of the RVOT, severe fetal edema, severe tricuspid regurgitation with low velocity (<2.5 m/s), and right ventricle-dependent coronary circulation. Two weeks after FCI, echocardiographic data were collected.

### 2.6 Data collection

Echocardiographic data from the first consultation and late-gestational follow-up were collected. No sex-based or race/ethnicity-based differences were included. Postnatal outcomes were classified as biventricular (BiV) circulation or non-BiV circulation. According to the established standards, BiV is defined as the RV being the only source of pulmonary blood flow (3, 18). It included individuals without an atrial septal defect or with an atrial septal defect shunting predominantly left-to-right and saturations of ≥92% at the latest follow-up (19). Non-BiV circulatory patterns included univentricular and one-and-a-half circulation.

### 2.7 Statistics

Continuous variables were expressed as mean ± standard deviation. Given the small sample size and potential variance inequality, Welch's *t*-test was performed. Before conducting the analyses, the normality of continuous variables was evaluated using the Shapiro–Wilk test and visual inspection (e.g., Q–Q plots). These assessments confirmed that the data approximated a normal distribution, supporting the appropriateness of using Welch's *t*-test for comparisons.

Categorical variables were presented as frequencies and percentages, and comparisons were performed using the chi-squared test. Statistical significance was set at a two-tailed *P* value <0.05. All analyses were conducted using SPSS software (version 26.0, IBM Corp., Armonk, NY, USA).

## 3 Results

### 3.1 Demographic features

Eighty-one patients with RV EFE, comprising 29 (35.8%) with CPS and 52 (64.2%) with PA, were identified. Among the 29 CPS patients, 23 (79.3%) were classified as Grade 1, while 6 (20.7%) were classified as Grade 2. Similarly, of the 52 PA patients, 43 (82.7%) were categorized as Grade 1, and 9 (17.3%) were categorized as Grade 2. There was no statistically significant difference in the distribution of RV EFE grades between CPS and PA groups (*P* = 0.707).

The severity of EFE did not exhibit significant changes over time, and further follow-up is needed. In addition, there were two fetuses with ventriculo-coronary arterial communication, both of which exhibited Grade 2 EFE. Unfortunately, both pregnancies were terminated, so they were not included in this study.

Ten of the 81 fetuses underwent FCI. The gestational age at FCI was 28.16 ± 0.35 weeks. Echocardiograms before FCI were compared with those of non-FCI fetuses. FCIs were considered technically successful if a balloon was inflated across the valve and there was improvement in anterograde flow without severe complications, as defined previously (3). Among the 10 fetuses, four (40%) experienced sustained bradycardia lasting ≥30 s after perforation. After the right atrial bolus of epinephrine, all foetal heart rates returned to normal. There were no cases of pericardial effusion requiring treatment during the FCI procedures.

### 3.2 Echocardiographic findings at initial consultation

The gestational age at initial diagnosis was 23.43 ± 0.92 weeks. By consensus, the severity of EFE was assessed as Grade 1 in 66 (81.5%) patients and Grade 2 in 15 (18.5%) patients. Among the 10 fetuses who underwent FCI, five (50%) were assessed as Grade 1 EFE, and five (50%) were Grade 2 EFE. Among the 71 fetuses who did not undergo FCI, 61 (85.9%) had Grade 1 EFE, and 10 (14.1%) had Grade 2 EFE.

The data in Table 2 show statistically significant differences in TV/MV, RV sphericity TV *z*-score and tricuspid regurgitation between the two groups. The Grade 2 EFE group had significantly greater RV sphericity values (RV short-axis to long-axis dimension) than did the Grade 1 EFE group (*P* < 0.001). Notably, greater RV sphericity implied more severe noncompliance and worse diastolic function.

**Table 2:** Anatomic characteristics on fetal echocardiography at initial consultation.

Variable	Grade 1 EFE (*n* = 66; 81.5%)	Grade 2 EFE (*n* = 15; 18.5%)	*P* value
GA (weeks)	23.50 ± 0.98	23.15 ± 0.58	0.196
RV/LV	0.63 ± 0.19	0.61 ± 0.17	0.702
PV/AV	0.78 ± 0.19	0.85 ± 0.17	0.143
TV/MV	0.89 ± 0.22	0.75 ± 0.15	**0**.**018**
RV sphericity	0.61 ± 0.11	0.73 ± 0.06	**<0**.**001**
TV *z*-score	0.79 ± 1.59	−0.21 ± 0.73	**0**.**001**
Tricuspid regurgitation			
None/Mild	42 (63.6%)	5 (33.3%)	**0**.**043**
Moderate/Severe	24 (36.4%)	10 (66.7%)	

EFE, endocardial fibroelastosis; GA, gestational age; RV/LV, right/left ventricular long-axis dimension; PV/AV, pulmonary/aortic valve annulus diameter; TV/MV, tricuspid/mitral valve annulus diameter.

Data presented as mean ± standard derivation. Right ventricle (RV) sphericity was estimated as the right ventricular short-axis to long-axis dimension ratio. A *P* value <0.05 is considered statistically significant and presented in bold.

The gestational age, RV/LV ratio, and PV/AV ratio did not significantly differ between the two groups (*P* > 0.05).

### 3.3 Follow-up at late gestation

From the first consultation to the late-gestational follow-up, there were statistically significant differences in the changes in TV/MV (*P* = 0.042) and TV *z*-scores (*P* = 0.001) between the Grade 1 and Grade 2 RV EFE groups (Table 3).

**Table 3:** Changes in anatomic characteristics from initial consultation to late gestation follow-up.

Rate of change in variable (per week)	Grade 1 EFE (*n* = 66; 81.5%)	Grade 2 EFE (*n* = 15; 18.5%)	T value	*P* value
Duration (days)	49.05 ± 10.89	44.86 ± 10.35	1.400	0.176
RV/LV	0.006 ± 0.016	−0.002 ± 0.007	1.962	0.053
PV/AV	−0.003 ± 0.011	−0.008 ± 0.020	0.932	0.365
TV/MV	0.000 ± 0.026	−0.018 ± 0.021	2.069	**0**.**042**
RV sphericity	−0.005 ± 0.026	0.001 ± 0.009	−0.893	0.375
TV *z*-score	−0.008 ± 0.156	−0.105 ± 0.070	3.673	**0**.**001**

EFE, endocardial fibroelastosis; RV/LV, right/left ventricular long-axis dimension; PV/AV, pulmonary/aortic valve annulus diameter; TV/MV, tricuspid/mitral valve annulus diameter.

Data presented as mean ± standard derivation. Right ventricle (RV) sphericity was estimated as the right ventricular short-axis to long-axis dimension ratio. A *P* value <0.05 is considered statistically significant and presented in bold.

Among the 10 FCI fetuses, the RV/LV ratio, RV sphericity and TV *z*-score were restored more significantly in Grade 2 EFE fetuses than in Grade 1 EFE fetuses (Table 4). The results indicate that FCI has certain potential to improve circulatory development in fetuses with Grade 2 EFE. The Grade 2 RV EFE was suggested to be a putative indicator of FCI.

**Table 4:** Changes from initial consultation to preintervention and changes from preintervention to 2-weeks later follow-up in 10 fetal cardiac intervention (FCI) cases.

Rate of change in variable (per week)	Grade 1 EFE (*n* = 5)			Grade 2 EFE (*n* = 5)		
	Before FCI	After FCI	*P* value	Before FCI	After FCI	*P* value
RV/LV	0.007 ± 0.011	0.055 ± 0.026	**0**.**010**	−0.004 ± 0.008	0.072 ± 0.019	**<0**.**001**
PV/AV	−0.001 ± 0.017	0.041 ± 0.024	**0**.**013**	−0.009 ± 0.015	0.006 ± 0.010	0.088
TV/MV	0.030 ± 0.032	0.049 ± 0.050	0.492	−0.001 ± 0.012	0.029 ± 0.036	0.144
RV sphericity	0.051 ± 0.030	−0.015 ± 0.020	**0**.**004**	0.003 ± 0.007	−0.066 ± 0.010	**<0**.**001**
TV *z*-score	−0.165 ± 0.163	−0.010 ± 0.181	0.192	−0.232 ± 0.084	0.286 ± 0.206	**0**.**001**

EFE, endocardial fibroelastosis; RV/LV, right/left ventricular long-axis dimension; PV/AV, pulmonary/aortic valve annulus diameter; TV/MV, tricuspid/mitral valve annulus diameter.

Data presented as mean ± standard derivation. Right ventricle (RV) sphericity was estimated as the right ventricular short-axis to long-axis dimension ratio. A *P* value <0.05 is considered statistically significant and presented in bold.

In Grade 2 EFE cases, fetuses who underwent FCI had greater changes in RV/LV, TV/MV and RV sphericity than did non-FCI fetuses (Table 5). These results indicated that FCI benefited Grade 2 EFE fetuses by restoring the development of ventricular structure.

**Table 5:** Difference of the changes in grade 2 right ventricular (RV) endocardial fibroelastosis (EFE).

Rate of change in variable (per week)	Non-FCI (*n* = 10)	FCI (*n* = 5)	T value	*P* value
RV/LV	−0.001 ± 0.007	0.072 ± 0.019	−8.438	**0**.**001**
PV/AV	−0.007 ± 0.023	0.006 ± 0.010	−1.236	0.238
TV/MV	−0.018 ± 0.021	0.029 ± 0.036	−2.663	**0**.**042**
RV sphericity	−0.000 ± 0.010	−0.066 ± 0.010	11.983	**<0**.**001**
TV *z*-score	−0.117 ± 0.050	−0.080 ± 0.101	−0.772	0.475

FCI, fetal cardiac intervention; RV/LV, right/left ventricular long-axis dimension; PV/AV, pulmonary/aortic valve annulus diameter; TV/MV, tricuspid/mitral valve annulus diameter.

Data presented as mean ± standard derivation. RV sphericity was estimated as the right ventricular short-axis to long-axis dimension ratio. A *P* value <0.05 is considered statistically significant and presented in bold.

### 3.4 Postnatal circulatory outcomes

**Figure 2:**
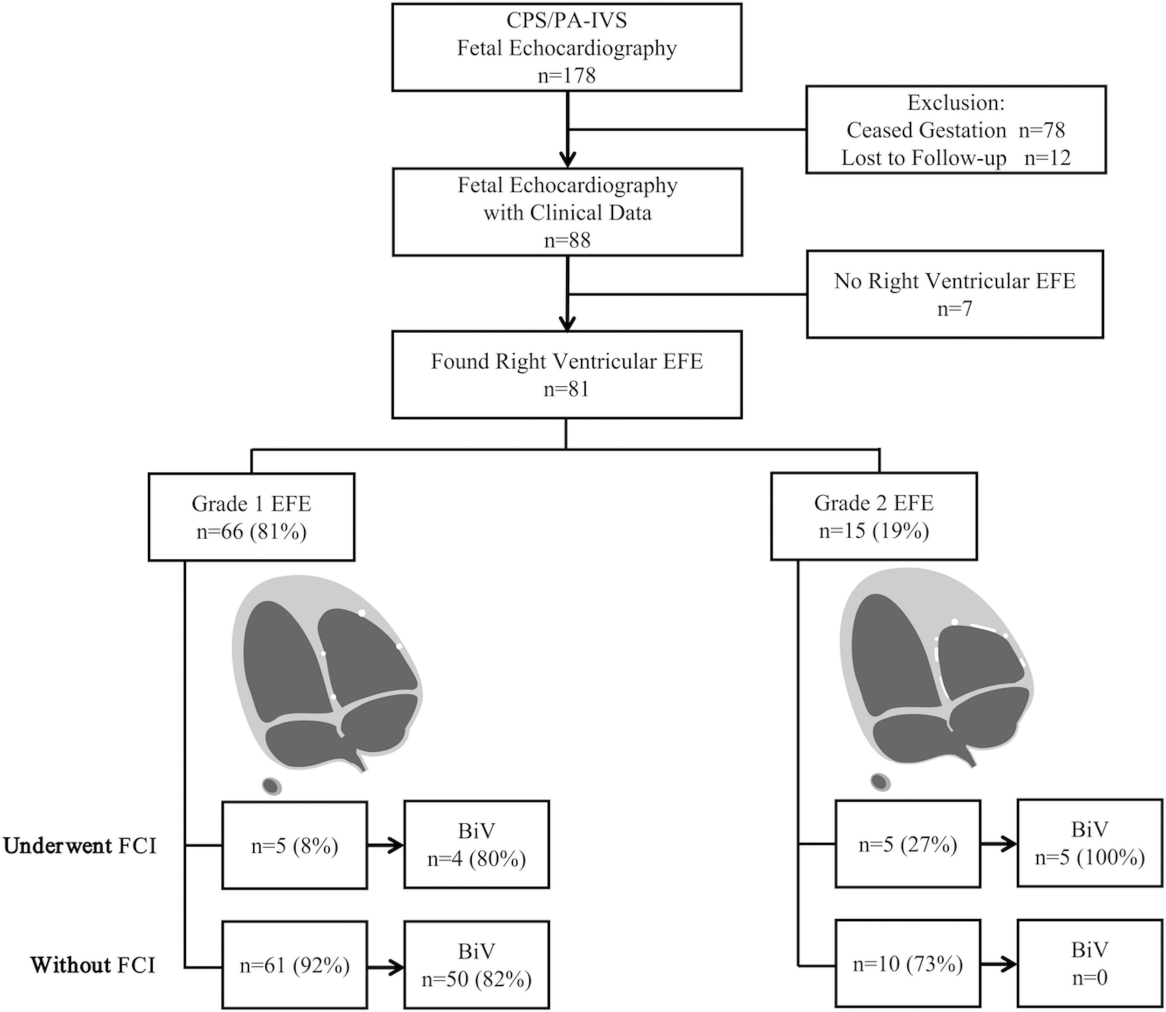
Flowchart of right ventricular endocardial fibroelastosis (EFE) gradation and postnatal outcomes. BiV, biventricle; CPS/PA-IVS, critical pulmonary stenosis and pulmonary atresia with intact ventricular septum; FCI, fetal cardiac intervention.

The average follow-up time was 32 (6–50) months. The circulatory state was assessed at the time of discharge, 28 days of life, and 1 year of age. By the end of follow-up, 59 (72.8%) patients achieved BiV circulation, including 9 who underwent FCI and 50 who did not (Figure 2). The circulatory outcomes and echocardiographic characteristics at initial consultations in FCI and non-FCI patients are shown in Supplementary Table 1 and Supplementary Table 2. Moreover, among the 81 patients, the severity of EFE did not significantly change over time.

Among the 10 FCI fetuses, 9 (90%) achieved BiV circulation. Specifically, four had Grade 1 EFE, and five had Grade 2 EFE. One patient diagnosed with PA and Grade 1 EFE underwent FCI but did not achieve a BiV outcome, probably due to the relatively severe hypoplasticity of the RV and valves (RV/LV = 0.47, PV/AV = 0.65, TV/MV = 0.76, RV sphericity = 0.43, and TV *z*-score = −1.17).

Among the 71 non-FCI fetuses, 50 (70.4%) achieved BiV circulation, and all had Grade 1 EFE. BiV was not achieved in twenty-one (29.6%) non-FCI fetuses. Specifically, 11 had Grade 1 EFE, and 10 had Grade 2 EFE.

## 4 Discussion

In this study, we defined two grades of RV EFE in CPS/PA-IVS fetuses. To the best of our knowledge, this is the first report regarding the grading of RV EFE severity in RVOT obstruction-related diseases. The results revealed that RV sphericity values were greater in the Grade 2 EFE group than in the Grade 1 EFE group, indicating an association between EFE severity and RV sphericity. As the normal RV is elliptical, stiffness and hypoplasia of the RV might manifest as increased sphericity (20).

RV EFE severity correlated with changes in the TV *z*-score and TV/MV from the initial consultation to late-gestational follow-up in patients with or without FCI. Regarding FCI fetuses, the Grade 2 EFE group showed more significant changes in the TV *z*-score, TV/MV, and RV/LV from before to after FCI than did the Grade 1 EFE group.

RV EFE severity was also associated with postnatal outcomes. Without successful FCIs, individuals with Grade 2 EFEs were more likely to achieve non-BiV circulation. In contrast, foetuses with Grade 1 EFE had a more favourable prognosis and were more likely to achieve biventricular outcomes without FCI.

The presence and severity of EFE have been suggested as inclusion criteria for intrauterine balloon valvuloplasty in fetuses with critical aortic stenosis, and they are related to the possibility of a biventricular outcome following prenatal intervention for fetal aortic stenosis with evolving HLHS (15). In the context of PA-IVS, the hypothesis regarding the development of EFE is based on elevated pressure within the right ventricle (21). However, systematic assessments of RV EFE have not been reported.

The goal of FCI is to enhance the possibility of biventricular circulation to improve long-term outcomes. Ventricular and valvular hypoplasia, fibrosis, and abnormalities in coronary arteries are prominent predictors of poor outcomes in individuals with CPS/PA-IVS. The current study demonstrated that successful FCI could halt the progression of RV hypoplasia in fetuses with Grade 2 EFE. It has been proven that in the early stages of LV outflow tract disorders, fibrotic alterations are reversible (22). Since fibrosis in the setting of RV hypertension is known to be partially reversible in postnatal congenital heart disease (such as tetralogy of Fallot), cautiously indicated and timed prenatal intervention may prevent the development of excessive EFE, which could otherwise seriously impair RV function and result in single ventricular circulation and a poor prognosis (23). FCI should be considered if there is any potential to restore RV development and preserve a biventricular outcome. Furthermore, FCI has demonstrated benefits in improving patient prognosis (5, 23, 24). Nevertheless, the current FCI criteria for CPS/PA-IVS are still inconsistent and contradictory among institutions (25).

FCI candidate selection is critical and challenging due to the limited population and scarcity of data. Most institutions base their decisions on geometric and morphological indicators, such as RV structures with respect to left-sided structures, and indications of RV preload and pressure-generating capacity (4, 25). Despite meeting the current FCI criteria, some patients achieved biventricular outcomes without FCI (7). Due to the limited population of CPS/PA-IVS patients and restricted examination technology, pathophysiologic variables are rarely assessed before FCI. Thus, to more accurately assess FCI, we aimed to identify a new predictor. Fortunately, Grade 2 RV EFE was found to be a potent indicator for FCI.

Although RV EFE was identified in this study as a potential indicator for FCI in CPS/PA-IVS, its impact on prognosis has been rarely reported due to the extremely low prevalence of these conditions. Roman et al. (26) reported that RV EFE and/or RV sinusoids were observed in 20% of all fetuses; however, only RV sinusoids were exclusively associated with non-biventricular outcomes. Notably, this study did not describe the severity of RV EFE or explore its implications for intervention. Currently, no other studies have systematically evaluated the grading of RV EFE severity or its potential role in guiding FCI. Our study bridges this gap by demonstrating that Grade 2 RV EFE may serve as a potential indicator for FCI, highlighting the importance of echocardiographic stratification in clinical decision-making. These findings provide a foundation for future research to further investigate the clinical significance of RV EFE in fetal cardiac interventions.

Clarifying the theoretical mechanisms and natural progression of CPS/PA-IVS is crucial for guiding appropriate patient selection and timing for FCI. In the developing heart, EFE secondary to altered hemodynamics has been discovered (20). EFE is directly associated with flow disruptions, resulting in an infiltrative growth pattern that enhances the diastolic stiffness of the RV. Endothelial-to-mesenchymal transition (EndMT) has been demonstrated to be one underlying mechanism of EFE formation. Endothelial cells of the endocardial layer convert into fibroblasts as a consequence of EndMT (27). Patients with stenotic or defective valves that cause flow disturbances in the ventricles develop fibrotic tissue covering the ventricle resulting from EndMT. Thus, EndMT may be a potential therapeutic target.

There are several limitations in the present study. First and foremost, the integrative effect of EFE together with other predictors on postnatal outcomes remains unclear. Additionally, the accuracy of the echocardiographic assessment of EFE is not flawless (28, 29). As this study was retrospective, the scanning modalities were not specifically optimized for assessing fibroelastosis. Additionally, the follow-up duration was relatively short. Although the short-term data are promising, further study is necessary for long-term data. Future studies will specifically focus on determining the prevalence of CPS/PA-IVS and the incidence of RV EFE.

In summary, for the first time, we graded RV EFE in CPS/PA-IVS and revealed the potential of FCI to preserve the cardiac development of Grade 2 RV EFE fetuses. Further study is warranted to integrate EFE with other predictors to select FCI candidates more appropriately.

## Data Availability

The original contributions presented in the study are included in the article/Supplementary Material, further inquiries can be directed to the corresponding author.
