# Supplementary material for: Assessment of right ventricular endocardial fibroelastosis in fetuses with critical pulmonary stenosis and pulmonary atresia with intact ventricular septum
Source: Front Pediatr. 2025 Jan 10;12:1518898. doi: 10.3389/fped.2024.1518898 (PMC11757882; doi:10.3389/fped.2024.1518898)
Supplement: Supplementary file 1 [file Datasheet1.pdf]

Supplemental Material

1 Supplementary Figure

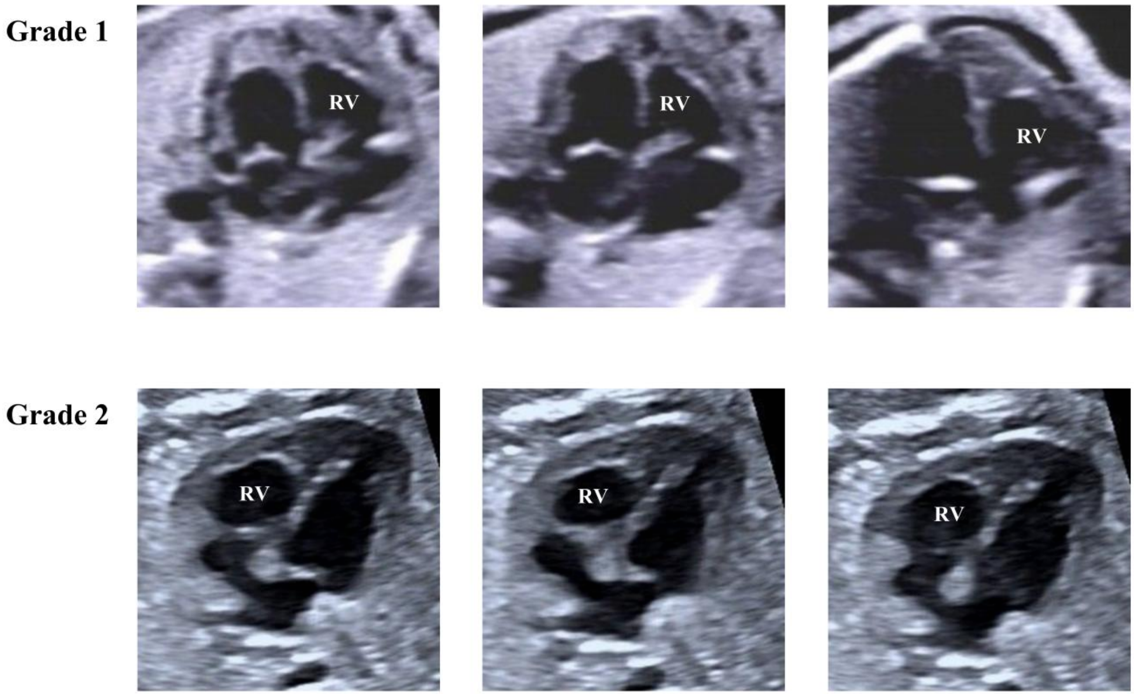

Supplemental Figure 1 Echocardiographic images demonstrating examples of right ventricle (RV) endocardial fibroelastosis grades. Three images from the same scan were used for each grade illustration.

2 Supplementary Tables

Supplemental Table 1. Circulatory outcomes and echocardiographic characteristics of the 10 FCI cases.

	BiV (n=9; 90%)	Non-BiV (n=1; 10%)
GA (weeks)	23.71 (22.43-26.14)	24.00
RV/LV	0.71 (0.57-0.79)	0.47
PV/AV	0.80 (0.56-1.00)	0.65
TV/MV	0.78 (0.57-0.85)	0.76
RV sphericity	0.65 (0.33-0.88)	0.43
RV EFE: Grade 1, 2	4, 5	1, NA

Data presented as median (minimum-maximum) or count. Right ventricle (RV) sphericity was estimated as the right ventricular short-axis to long-axis dimension ratio. *BiV:* biventricle, *GA:* gestational age, *RV EFE:* right ventricular endocardial fibroelastosis, *RV/LV:* right/left ventricular long-axis dimension, *PV/AV:* pulmonary/aortic valve annulus diameter, *TV/MV:* tricuspid/mitral valve annulus diameter

Supplemental Table 2. Circulatory outcomes and echocardiographic characteristics of the 71 non-FCI cases.

	BiV (n=50; 70%)	Non-BiV (n=21; 30%)
GA (weeks)	22.57 (22.29-26.14)	23.14 (22.29-25.43)
RV/LV	0.67 (0.24-1.28)	0.50 (0.35-0.92)
PV/AV	0.82 (0.33-1.37)	0.73 (0.45-1.17)
TV/MV	0.92 (0.70-1.23)	0.76 (0.30-0.98)
RV sphericity	0.66 (0.40-0.80)	0.71 (0.46-0.78)
RV EFE: Grade 1, 2	50, 0	11, 10

Data presented as median (minimum-maximum) or count. Right ventricle (RV) sphericity was estimated as the right ventricular short-axis to long-axis dimension ratio. *BiV:* biventricle, *GA:* gestational age, *RV EFE:* right ventricular endocardial fibroelastosis, *RV/LV:* right/left ventricular long-axis dimension, *PV/AV:* pulmonary/aortic valve annulus diameter, *TV/MV:* tricuspid/mitral valve annulus diameter
